# Neuroscience Has the Power to Change the Criminal Justice System

**DOI:** 10.1523/ENEURO.0362-16.2016

**Published:** 2017-01-27

**Authors:** Cara M. Altimus

**Affiliations:** 2015/2016 Science and Technology Policy Fellow, American Association for the Advancement of Science, Washington, DC 20005

**Keywords:** criminal justice, incarceration, science policy

## Abstract

As a neuroscientist working in the Department of Justice for the past year, I observed that many of the challenges of crime and justice have solutions rooted in our understanding of neuroscience. However, the neuroscience community seems absent from conversations regarding these solutions.

## Significance Statement

Criminal justice policy-makers, locally and globally, struggle to develop and implement policies to balance justice, punishment, and rehabilitation. However, many of the issues that criminal justice policy is tasked to manage involve the interactions of people and the modification of behavior. I spent a year working as a scientist in the Department of Justice and had the privilege of understanding what the biggest problems facing policy-makers currently are and how neuroscientists can contribute to better policy-making. This article is a call to neuroscientists and criminal justice policy-makers to bridge the knowledge gap and incorporate our understanding of the brain into future policies.

## Introduction

In 2015, the Bureau of Justice Statistics reported that nearly 7 million people were incarcerated or under the supervision of the criminal justice system in the United States. Of those incarcerated, the Department of Justice (DOJ) estimates that more than half have mental health problems and two-thirds report substance use or dependence. Those working in the DOJ and within the criminal justice system—both in the United States and internationally—are addressing many human behavior issues and asking questions relevant to neuroscience. Some of the questions are neurodevelopmental: When does the brain reach maturity and become an “adult” brain? Do all human brains mature at the same rate? Is the rate the same for males and females?

Other questions focus on stress and trauma. Does childhood trauma affect decision-making and violent behavior later in life? For police officers, how does stress, such as that encountered at a public demonstration, affect their performance, decision-making, and bias? And does the acute trauma of an event affect eyewitness testimony? If it does, then how?

Some of the questions are related to finding practical solutions to real problems. What treatments do specific substance use disorders best respond to? How can experts quantitatively assay stress levels in corrections and police staff? What could be done to ameliorate the impact of stress once it is detected?

These questions are similar to questions that are also researched in the halls of university neuroscience departments, yet a gap remains between academic neuroscience and criminal justice researchers.

## Discussion

While those working in criminal justice struggle for answers to behavioral issues, we in the neuroscience communities know that genetics and experience affect neural development, and that trauma and stress impact decision-making, as does sleep and fatigue. Importantly, neuroscientists spend a great deal of time thinking about why these changes in the brain lead to changes in behavior, and what interventions could be most useful. Neuroscientists do not think twice when asked if the brain controls all human behavior, whether the use of illicit substances changes neural circuits, or whether isolation in solitary confinement affects a person’s mental health. These questions have all been addressed in neuroscience research and solutions based on these scientific findings are relevant across global boarders.

In the same ways that neuroscience research is applied to clinical psychiatry, it should be translated and applied to criminal justice policy and practice. As a society, we need to understand and explain the underlying biological systems controlling the complex behaviors that the criminal justice system must deal with. We need neuroscience researchers to work with criminal justice institutions to better understand the causes and effects of criminal behavior, and how criminal justice policy and practice can be improved to not only do the least harm, but also do the most good.

Neuroscience has advanced to a point where it can make a significant difference in the brain-based issues that the criminal justice system commonly encounters. Neuroscientists have gained the understanding and developed the techniques that allow them to image neural activity in humans during behavior. Researchers can turn off and on specific cell types to modify behavior in animals, work that has implications for understanding the neural basis of human behavior.

Neuroscience researchers are actively mapping circuits and the changes involved in addiction, violence, and mental illness. They are developing neurological devices to help those suffering from mental illness or substance use disorders when traditional pharmaceutical therapeutic methods do not work. Given these and other advances, we need to bring modern neuroscience to the criminal justice community. Funding agencies and organizations looking for solutions to criminal justice problems should include neuroscientists. And neuroscientists should look beyond the laboratories and clinics where we typically work and consider the positive impact our research could have in the criminal justice system. We can do this by working with criminal justice institutions and social service agencies to understand knowledge gaps and promote relevant research.

As neuroscientists, our understanding of one of the most complex biological systems known has the potential to change the way the justice system operates. Although it may go against the scientific norm of distance and neutrality, society as a whole, and the criminal justice system in particular, would benefit from active collaboration between the neurological sciences and the criminal justice system. The payoff for all of us could reach as far as it has in traditional medicine.

## Synthesis

Reviewing Editor: Christophe Bernard, INSERM & Institut de Neurosciences des Systèmes Decisions are customarily a result of the Reviewing Editor and the peer reviewers coming together and discussing their recommendations until a consensus is reached. When revisions are invited, a fact-based synthesis statement explaining their decision and outlining what is needed to prepare a revision will be listed below. The following reviewer(s) agreed to reveal their identity: NONE. Note: If this manuscript was transferred from JNeurosci and a decision was made to accept the manuscript without peer review, a brief statement to this effect will instead be what is listed below.

NA

